# Efficacy of restrictive versus liberal transfusion strategies in patients with traumatic brain injury: a systematic review and meta-analysis of randomized controlled trials

**DOI:** 10.1186/s13613-024-01411-1

**Published:** 2024-11-28

**Authors:** Xiang Yuan, Sen Zhang, Jun Wan, Cheng Chen, Peng Wang, Shijie Fan, Yuyang Liu, Jingxian Yang, Jiayi Hou, Qiaoyu You, Xiao Li, Kuilin Li, Ziyan Xiang, Yang Rao, Yu Zhang

**Affiliations:** 1 Department of Neurosurgery, Affiliated Hospital of Chengdu University, Chengdu, Sichuan China; 2 Department of critical care medicine, Affiliated Hospital of Chengdu University, Chengdu, Sichuan China; 3 Center for Evidence-based Medicine, Affiliated Hospital of Chengdu University, Chengdu, Sichuan China; 4 Department of Neurosurgery, West China Hospital, Sichuan University, Chengdu, Sichuan China; 5 Clinical Medical College, Affiliated Hospital of Chengdu University, Chengdu, Sichuan China

**Keywords:** Traumatic brain injury, Transfusion, Meta-analysis

## Abstract

**Background:**

The effects of restrictive versus liberal transfusion strategies in critically ill patients with traumatic brain injury (TBI) and anemia, particularly in adult patients with moderate to severe TBI, remain inconclusive. Therefore, this systematic review and meta-analysis aim to evaluate the comparative impact of restrictive and liberal red blood cell transfusion strategies among critically ill adult patients with moderate to severe TBI.

**Methods:**

We conducted a search of PubMed, EMBASE, and the Cochrane Central Register of Controlled Trials from their inception through October 20, 2024, to identify randomized controlled trials that compared restrictive (transfusions at a hemoglobin level of ≤ 7 g/dL) and liberal (transfusions at a hemoglobin level of ≤ 9–10 g/dL) transfusion strategies in adult patients with TBI. The primary outcome was mortality, with secondary outcomes including an unfavorable neurological outcome at six months, as determined by the Glasgow Outcome Scale (GOS < 4; or Glasgow Outcome Scale-Extended [GOSE] < 6), and the number of units of packed red blood cells (pRBCs) transfused.

**Results:**

Five randomized controlled trials involving 1,528 patients were included in the analysis. The results showed that restrictive transfusion, compared to liberal transfusion, had no impact on mortality (RR 1.00, 95% CI 0.80 to 1.24, I^2^ = 0%) or unfavorable neurological outcome at 6 months (RR 1.06, 95% CI 0.94 to 1.20, I^2^ = 47%). Restrictive transfusion was associated with a reduction in the number of units of pRBCs transfused (MD -2.62, 95% CI -3.33 to -1.90, I^2^ = 63%).

**Conclusion:**

In patients with TBI, a restrictive transfusion strategy did not reduce the risk of mortality or unfavorable neurological outcome compared with a liberal transfusion strategy.

**Supplementary Information:**

The online version contains supplementary material available at 10.1186/s13613-024-01411-1.

## Introduction

Anemia is a common complication in critically ill patients with traumatic brain injury (TBI), and it is associated with unfavorable neurological outcomes and elevated mortality rates in patients with TBI requiring intensive care unit admission [1, 2]. Red blood cell (RBC) transfusions are frequently used to treat anemia, yet their use in this population can increase the risk of infections and transfusion-related acute lung injury [2–4]. Clinical guidelines and reviews comparing the outcomes of liberal and restrictive transfusion strategies suggest that a restrictive approach is safe in most settings [5, 6]. However, these guidelines caution that current evidence is insufficient to guide transfusion practices in patients with TBI, as restrictive strategies may not meet the heightened metabolic demands of the cerebral parenchyma, potentially affecting its oxygen delivery capacity and neuronal functionality [5, 6].

Previous meta-analysis on TBI has explored the impacts of transfusions under either restrictive or liberal strategies have yielded inconclusive results, as they were primarily based on observational studies, which may introduce bias [7]. Recently, the emergence of two large-scale randomized controlled trials (RCTs) has provided substantial additional data, enhancing the overall sample size significantly [8, 9]. Now, we’re conducting a systematic review and meta-analysis of RCTs to compare the efficacy of two transfusion thresholds in patients with TBI.

## Methods

### Protocol and guidance

This systematic review was carried out in accordance with the Preferred Reporting Items for Systematic Reviews and Meta-Analyses (PRISMA) guidelines [10]. Moreover, prior to initiating the study, we prospectively registered the research protocol at PROSPERO with the registration number CRD42024574035. This step was implemented to enhance transparency and adherence to established standards in evidence-based research.

### Search methods and selection criteria

Studies were included if they (1) enrolled patients with moderate to severe TBI; (2) compared restrictive transfusion strategies with liberal transfusion strategies (restrictive transfusion strategies were defined as transfusions at a hemoglobin level of ≤ 7 g/dl, while liberal transfusion strategies were defined as transfusions at a hemoglobin level of ≤ 9–10 g/dl); (3) were RCTs; and (4) reported at least one of the predefined outcome measures of interest, such as mortality, unfavorable outcome.

### Outcomes

The primary outcome was mortality at 6 months. Secondary outcomes included an unfavorable neurological outcome at 6 months, assessed using the Glasgow Outcome Scale (GOS < 4) or the Glasgow Outcome Scale-Extended (GOSE < 6) [11], and the total number of packed red blood cell units transfused. The number of pRBC units transfused was considered to capture the differences in blood product utilization and potential transfusion-related risks between restrictive and liberal transfusion strategies.

### Information sources and search strategy

We searched PubMed, EMBASE, and the Cochrane Central Register of Controlled Trials from inception until October 20, 2024. The search equation includes free terms and MeH terms incorporating these keywords: “traumatic brain injury,” “anemia,” “blood transfusion,” “erythrocyte transfusion,” and “blood component transfusion.” There were no limitations based on language. Table S1 in the Data Supplement outlines the comprehensive search terms employed in each database searched.

### Study inclusion

Two reviewers (XY and SZ) independently evaluated all titles and abstracts identified through the systematic search. To ensure accuracy, a third reviewer (YZ) checked for any discrepancies in the collected data. Any disagreements were resolved through discussion or adjudicated by a third reviewer (YZ).

### Data extraction

Two reviewers (XY and SZ) independently extracted data on the characteristics of the included trials, including the study author, publication year, study methodology, research aim, hemoglobin threshold (in grams per deciliter), patient demographics, and presentation attributes such as age and gender. To ensure accuracy, a third reviewer (YZ) reviewed the extracted data for any errors. Disagreements between reviewers were resolved through discussion.

### Assessment of the risk of bias

Two reviewers (XY and SZ) independently assessed the risk of bias in the trials using the Cochrane Risk of Bias-2 tool [12] across five domains. Each domain in all trials was assigned a study-level score indicating the level of bias risk: low, high, or unclear. Disagreements between reviewers were resolved through discussion. If consensus could not be reached, a final judgment was provided by a third author (YZ).

### Confidence of evidence

Two authors (XY and SZ) independently assessed the quality of evidence for primary and secondary outcomes using the Grading of Recommendation, Assessment, Development and Evaluation (GRADE) [13]. The quality of evidence was categorized as high, moderate, low, or very low based on multiple factors including the evaluation of study design, risk of bias, inconsistency, imprecision, and indirectness of the included trials.

### Data analysis

We performed the statistical analysis utilizing Review Manager (version 5.4, The Cochrane Collaboration). For binary results, we computed the relative risk (RR) with 95% confidence intervals (CI). To assess continuous results, we calculated the mean difference (MD) with a 95% CI. Study heterogeneity was assessed using Cochrane’s Q and Higgins’ I^2^ statistics, with Q statistic values at a 10% level of significance (*p* < 0.1) and I^2^ values > 50% indicating significant heterogeneity. To ensure the validity of the findings, we utilized random-effect models for all outcomes. We considered a predefined two-sided *p*-value < 0.05 as statistically significant. Forest diagrams were constructed to illustrate the magnitude of effects and combined estimations. All statistical analyses were conducted using two-tailed tests, with a significance threshold of *p* < 0.05 indicating a statistically significant association. In this systematic review and meta-analysis, we did not pool individual patient data.

### Publication bias

If a meta-analysis included 10 or more studies, we would generate a funnel plot and perform quantitative assessments utilizing the Egger’s [14] and Begg’s tests [15] to comprehensively assess any potential biases associated with small study effects.

### Subgroup analysis

We conducted subgroup analysis on the primary outcome based on follow-up time, hemoglobin at randomization, and mean age.

### Sensitivity analysis

We performed a sensitivity analysis utilizing the following approaches: (1) utilizing a fixed-effect model; (2) excluding trials with a weight less than 10%; (3) excluding each trial individually.

## Results

**Fig. 1:**
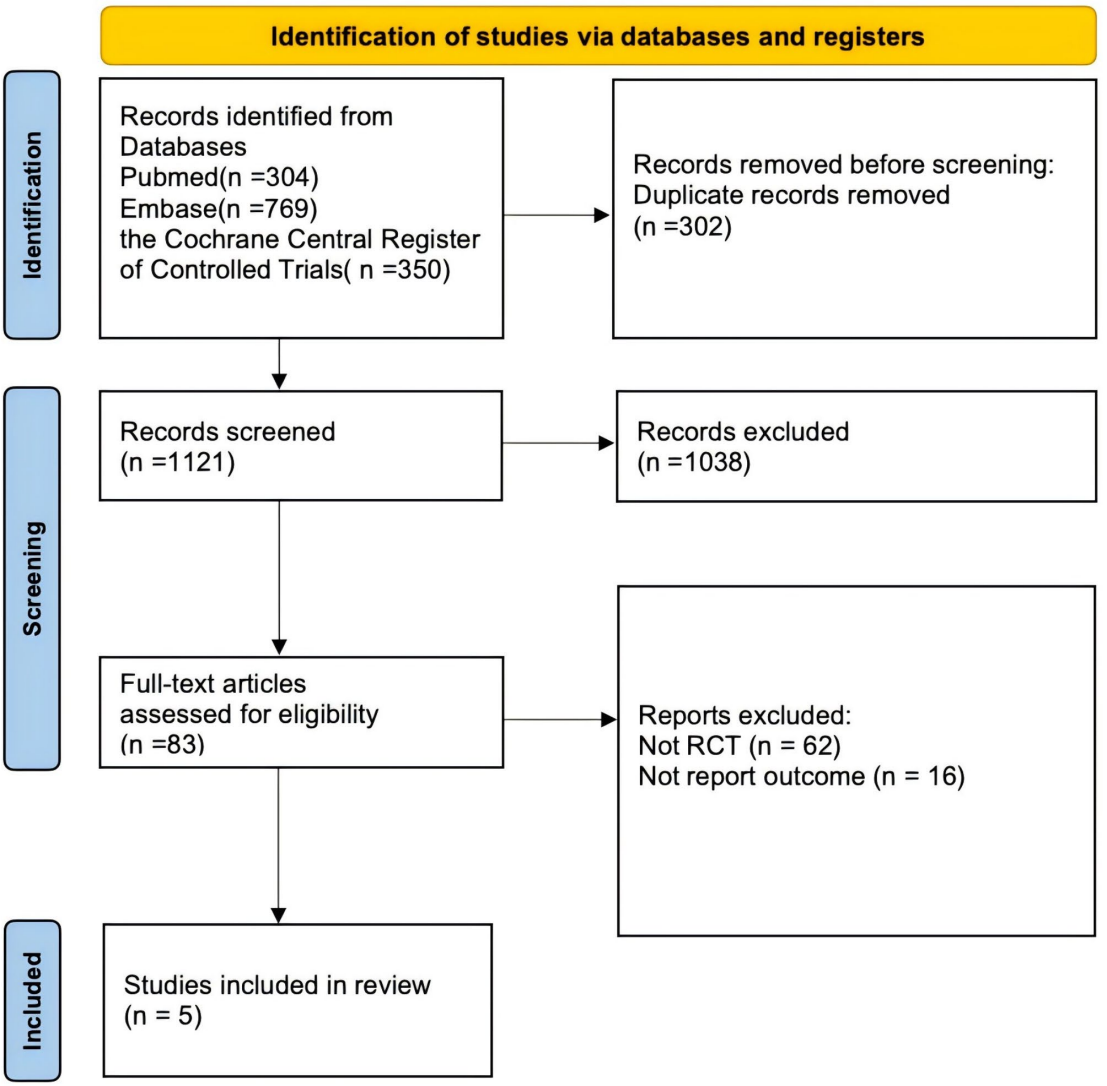
Search strategy and final included and excluded studies

Our search strategy initially identified 1423 records. After removing duplicates, we screened a total of 1121 unique records. We then systematically evaluated the titles and abstracts of these records, followed by an in-depth assessment of the full texts of potentially eligible studies. Ultimately, we identified five trials [8, 9, 16–18] that satisfied the inclusion criteria for this systematic review (Fig. 1).

Our systematic review and meta-analysis included five RCTs [8, 9, 16–18], with four [8, 16–18] focusing on TBI and one [9] studying acute brain injury that included TBI. The trials were published between 2006 and 2024, and the sample size ranged from 44 to 742 patients (Table 1).

**Table 1:** Characteristics of studies included in the meta-analysis

Study	Mean age, years	Patients, *N*	Female, no. (%)	Transfused Threshold, g/dl		Glasgow Coma Scale: median (IQR)		Injury Severity Score: mean ± SD or median (IQR)		Hemoglobin at Randomization, g/dl: mean ± SD or median (IQR)		Units of Packed Red Blood Cells Transfused: mean ± SD	
				Liberal group	Restrictive group	Liberal group	Restrictive group	Liberal group	Restrictive group	Liberal group	Restrictive group	Liberal group	Restrictive group
McIntyre et al., 2006[17]	41	67	26	≤ 10	≤ 7	7 (5–9)	8 (5–10)	31 ± 13	30 ± 14	NR	NR	4.6 ± 2.5	1.4 ± 2.2
Robertson et al., 2014[18]	32	200	14	≤ 10	≤ 7	NR	NR	29 (25–35)	29 (25–38)	14.3 ± 2.0	14.3 ± 2.0	7.1 ± 5.3	4.7 ± 5.3
Gobatto et al., 2019[16]	35	44	9	≤ 9	≤ 7	5 (3–7)	5 (3–7)	28 ± 9	31 ± 9	7.9 ± 0.6	8.2 ± 1.0	3.1 ± 1.6	1.5 ± 1.7
Turgeon et al., 2024[8]	49	742	27	≤ 10	≤ 7	4 (1–5) *	4 (1–5) *	30 ± 11	32 ± 11	9.1 ± 0.8	9.1 ± 0.8	3.4 ± 2.2	0.4 ± 0.7
Taccone et al.,2024[9]	NR	475	NR	≤ 9	≤ 7	NR	NR	NR	NR	NR	NR	NR	NR

GCS: Glasgow Coma Scale, SD: Standard Deviation, IQR: Inter-Quartile Range, NR: not reported, * Glasgow Coma Scale motor score

Figure S1 in the Data Supplement presents the Risk-of-bias assessments. One trial had low risk of bias [16], while four trials had some concerns [9, 17–19] (Figure S1 in the Data Supplement). The quality of evidence for the mortality was moderate, while the unfavorable neurological outcome and the units of packed red blood cells transfused were low, as evaluated by GRADE (Table 2).

**Table 2:** Summary of findings and strength of evidence

Outcome	NO. of patients (trials)		Relative Effect	Absolute effect estimates (per 1000)	Quality of the evidence
**Primary outcome**					
Mortality	1045 (4)	RR = 1.00; 95% CI (0.80 to 1.24)		0 (47 to 56)	Moderate ^a^
**Secondary outcomes**					
Unfavorable neurological outcome at 6 months	1422 (4)	RR = 1.06; 95% CI (0.94 to 1.20)		54 (69 to 169)	Low ^a, c^
Units of packed red blood cells transfused	1047 (4)	MD = -2.62; 95% CI (-3.33 to -1.90)		2.62 (1.9 to 3.33)	Low ^a, b^

CI: confidence interval; RR: risk ratio

^a^ We downgraded our assessment of the evidence one level for imprecision due to the limited number of patients

^b^ We downgraded our assessment of the evidence one level for inconsistency due to the serious I^2^ value of 63%

^c^ We downgraded our assessment of the evidence one level for inconsistency due to the moderate I^2^ value of 47%. This inconsistency was further highlighted by the sensitivity analysis, which showed a significant reduction in heterogeneity and a change in the result when one particular study was excluded

**Fig. 2:**
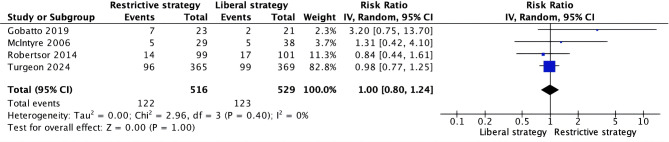
Forest plots comparing: mortality with restrictive and liberal transfusion thresholds in patients with traumatic brain injury. M–H: Mantel–Haenszel, CI: Confidence interval, df: Degrees of freedom

**Fig. 3:**
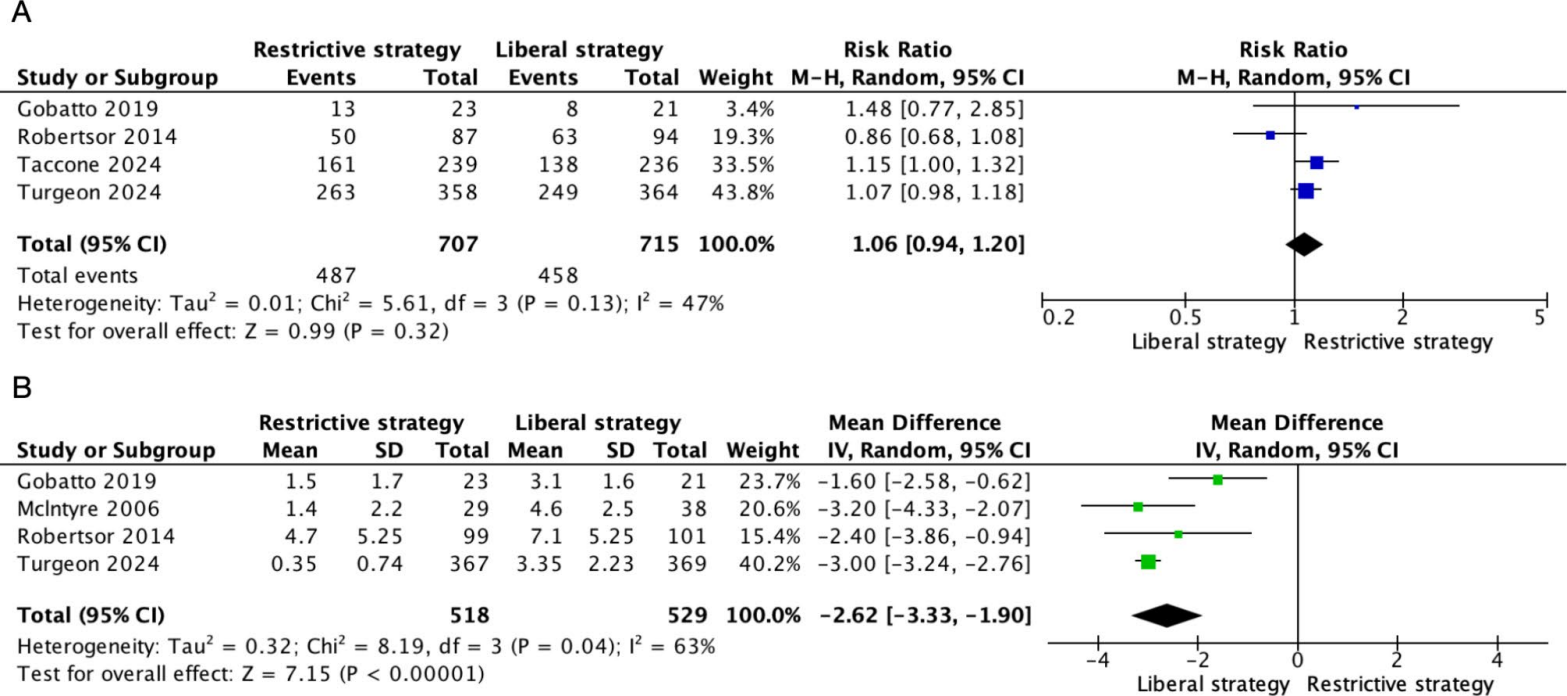
Forest plots comparing: (**A**) Unfavorable neurological outcome at six months (**B**) Units of packed red blood cells transfused. M–H: Mantel–Haenszel, CI: Confidence interval, df: Degrees of freedom

Four trials [16–19] reported on mortality, with no significant difference observed between restrictive and liberal transfusion thresholds (RR 0.80, 95% CI 0.80 to 1.24, I² = 0%) (Fig. 2). Four trials [9, 16, 18, 19] reported on the GOS or GOSE, with no significant difference in unfavorable neurological outcome at six months between the two transfusion thresholds (RR 1.06, 95% CI 0.94 to 1.20, I² = 47%) (Fig. 3A). Additionally, four trials [16–19] reported on the number of units of pRBCs transfused, with restrictive transfusion thresholds resulting in significantly fewer units being transfused (MD -2.62, 95% CI -3.33 to -1.90, I² = 63%) (Fig. 3B).

The robustness of mortality findings was confirmed through sensitivity analyses. Our sensitivity analyses of mortality revealed no significant differences in results, regardless of whether we employed a fixed-effect model, excluded trials with a weight of less than 10%, or excluded each trial individually (Table S2 in the Data Supplement). Conversely, when we excluded the trial by Robertson et al. (2014) [18], the effect on unfavorable neurological outcome became significant (Table S3 in the Data Supplement).

The results of the subgroups for mortality in patients with TBI indicated that none of the following factors significantly contributed to the source of heterogeneity: mean age (< 40 vs. >40, P for interaction = 0.61), follow-up time (two months vs. six months, P for interaction = 0.68), and mean hemoglobin at randomization (< 10 vs. >10, P for interaction = 0.42) (Figure S2 in the Data Supplement).

## Discussion

This systematic review and meta-analysis, which included five RCTs involving a total of 1528 participants, revealed that a restrictive transfusion strategy, compared to a liberal strategy, did not lead to a reduction in mortality or an unfavorable neurological outcome.

A previous meta-analysis [7] also examined this topic. Similar to our study, it similarly concluded that a restrictive transfusion strategy did not reduce mortality (odds ratio 0.94, 95% CI 0.60 to 1.48). However, that analysis included both trials and comparative observational studies, incorporating only three relatively small RCTs involving a total of 311 patients. In contrast, our comparative analysis was strictly focused on RCTs, including two newer large RCTs available in the literature, involving a total of 1528 patients. The data from these extensive studies served to strengthen the conclusions and enhance the precision of the treatment effects associated with a restrictive transfusion strategy.

Another meta-analysis of RCTs [20] encompassed a heterogeneous patient cohort with diverse neurocritical conditions (RR 1.42, 95% CI 0.42 to 4.78), including TBI, intracerebral hemorrhage, and subarachnoid hemorrhage. This broad scope has posed challenges in deriving precise implications for patients specifically with TBI. In contrast, our study exclusively investigated TBI patients, enabling us to deliver more tailored and concentrated outcomes for this distinct patient group. This focused methodology elevated the relevance and practicality of our conclusions for individuals with TBI.

The management of TBI patients involves a delicate balance between ensuring adequate blood flow and oxygenation to the brain, while minimizing the risks associated with blood transfusions. The debate between liberal and restrictive transfusion strategies in TBI patients is a critical aspect of this management, with each approach having its own set of advantages and disadvantages. Liberal transfusion, by setting a higher hemoglobin transfusion threshold (e.g., Hemoglobin (Hb) ≤ 10 g/dL), aims to maintain a sufficient Hb level to ensure adequate oxygen supply to the brain, potentially mitigating secondary brain injury due to anemia and improving neurological outcomes [21–23]. However, this approach may increase the risk of transfusion-related adverse events and complications, such as acute respiratory distress syndrome [24], transfusion-related acute lung injury [25], and infections, as well as consume more medical resources. On the other hand, restrictive transfusion, which adopts a lower Hb transfusion threshold (e.g., Hb ≤ 7 g/dL), seeks to reduce unnecessary transfusions and thus minimize transfusion-related risks and conserve medical resources. While this strategy has demonstrated advantages in mitigating transfusion-related hazards, it may also lead to inadequate blood supply to the brain, potentially impacting patient outcomes due to cerebral hypoxia [5, 26].

Hb concentration, while commonly used to guide transfusion decisions, reflects a measure of concentration in the blood rather than the actual volume of red blood cells. This limitation becomes critical when considering that TBI patients, especially those with intracranial hypertension, may require a more nuanced assessment of oxygen delivery to the brain [27]. Given that Hb concentration alone may not fully capture the adequacy of brain oxygen supply, alternative indicators such as jugular venous oxygen saturation and brain tissue oxygen monitoring can provide invaluable insights. These parameters can help inform transfusion decisions in situations where maintaining adequate brain oxygenation amidst elevated intracranial pressure is paramount [28–30]. A comprehensive approach that incorporates these alternative indicators, alongside considerations for the differences in intracranial pressure between moderate and severe TBI patients, is essential for optimizing treatment strategies and potentially improving patient outcomes while mitigating the risk of secondary brain injury. Future studies exploring optimal transfusion thresholds in TBI patients should consider incorporating these additional metrics to further refine and personalize transfusion strategies, ensuring that oxygen delivery to the brain is adequate and tailored to individual patient needs.

There are several limitations that need to be considered. First, the inconsistency in inclusion criteria, particularly regarding the definition of TBI, introduces variability into the research landscape. Second, variations in follow-up time among studies and characteristics of the patient population contribute to further heterogeneity. Variation in these parameters impart heterogeneity to a systematic review and meta-analysis of pooled data. To mitigate the impact of these disparities, we conducted stratified subgroup analyses. However, certain subgroup analyses, such as the severity of TBI could not be performed due to a lack of individual patient data from the component trials. Third, our primary outcome is primarily shaped by the HEMOTION trial [19]. While this enhances the statistical power of our analysis, it also means that our conclusions may be disproportionately affected by the characteristics and results of this particular study. Fourth, our primary outcome includes two very small RCTs which could potentially introduce bias. Consequently, we conducted a sensitivity analysis to assess the impact of these smaller studies on our findings. The results of these sensitivity analyses remain consistent with our findings, indicating that our conclusions are not significantly influenced by these smaller RCTs. Fifth, the limited number of RCTs included provided insufficient power to detect potential publication bias. Nevertheless, the likelihood of publication bias is minimal as most trials yielded neutral results in the primary outcome.

Additionally, our systematic review and meta-analysis did not demonstrate significant differences in mortality or unfavorable neurological outcome between restrictive and liberal transfusion strategies, this finding aligns with the results of the HEMOTION trial [19]. While HEMOTION was designed to detect a 10% absolute difference, the observed 5% difference suggests a non-significant trend favoring the liberal strategy, potentially due to inadequate power to detect smaller effect sizes, given the sample size [8, 31]. This underscores the complexity and uncertainty associated with the application of transfusion strategies in clinical practice. Therefore, while current evidence does not definitively support the superiority of one strategy over the other, these findings highlight the need for future research. Large-scale studies are warranted to investigate whether specific transfusion thresholds can provide meaningful clinical benefits for patients with TBI, further elucidating how to optimize transfusion strategies to improve patient outcomes.

## Conclusions

This systematic review and meta-analysis indicated that a restrictive transfusion strategy did not reduce the risk of mortality or unfavorable neurological outcome compared with a liberal transfusion strategy in patients with TBI. Nevertheless, current evidence does not definitively support the superiority of one strategy over the other. Further large-scale studies are warranted to determine whether specific transfusion thresholds provide clinically meaningful benefits in this patient population.

## Electronic supplementary material

Below is the link to the electronic supplementary material.

Supplementary Material 1

## Data Availability

All relevant data are within the manuscript and its Supporting Information files.

## Abbreviations

TBI: Traumatic brain injury
RBC: Red blood cell
Hb: Hemoglobin
GOS: Glasgow outcome scale
GOSE: Glasgow outcome scale-extended
GCS: Glasgow coma scale
pRBCs: Packed red blood cells
RCT: Randomized controlled trial
GRADE: Grading of recommendation, assessment, development and evaluation
PRISMA: Preferred reporting items for systematic reviews and Meta-analysis
CI: Confidence intervals
RR: Relative risk
OR: Odds ratio
MD: Mean difference
