# Supplementary material for: Efficacy of restrictive versus liberal transfusion strategies in patients with traumatic brain injury: a systematic review and meta-analysis of randomized controlled trials
Source: Ann Intensive Care. 2024 Nov 28;14:177. doi: 10.1186/s13613-024-01411-1 (PMC11604879; doi:10.1186/s13613-024-01411-1)

**Data Supplement**

| **Table S1.** Search strategy. |
| --- |
| **Table S2.** Summary of findings and strength of evidence. |
| **Table S3.** Sensitivity analysis of the primary outcome. |
| **Figure S1.** Risk of bias summary. |
| **Figure S2.** Subgroup analysis of the primary outcome. |

Table S1. Search strategy.

| PubMed | | |
| --- | --- | --- |
|  | Randomized Controlled Trial[Publication Type] | 624885 |
|  | Controlled Clinical Trial[Publication Type] | 715591 |
|  | randomized[Title/Abstract] | 727520 |
|  | randomly[Title/Abstract] | 445131 |
|  | trial[Title/Abstract] | 845736 |
|  | groups[Title/Abstract] | 2789638 |
|  | #1 OR #2 OR #3 OR #4 OR #5 OR #6 | 3999943 |
|  | Erythrocyte Transfusion[MeSH Terms] | 10353 |
|  | Blood Transfusion[MeSH Terms] | 94203 |
|  | Blood Component Transfusion[MeSH Terms] | 94203 |
|  | Hemoglobins[MeSH Terms] | 145372 |
|  | ((((((Erythrocyte Transfusion[Title/Abstract]) OR (Red Blood Cell Transfusion[Title/Abstract])) OR (Blood Transfusion[Title/Abstract])) OR (Blood Component Transfusion[Title/Abstract])) OR (Hemoglobin[Title/Abstract])) OR (Eryhem[Title/Abstract])) OR (Ferrous Hemoglobin[Title/Abstract]) | 207969 |
|  | Anemia[MeSH Terms] | 180597 |
|  | Anemia[Title/Abstract] | 143524 |
|  | #8 OR #9 OR #10 OR #11 OR #12 OR #13 OR #14 | 535479 |
|  | Brain Injuries, Traumatic[MeSH Terms] | 27489 |
|  | ((((((((Traumatic Brain Injuries[Title/Abstract]) OR (Brain Trauma[Title/Abstract])) OR (Brain Traumas[Title/Abstract])) OR (Traumatic Brain Injury[Title/Abstract])) OR (Traumatic Encephalopathies[Title/Abstract])) OR (Traumatic Encephalopathy[Title/Abstract])) OR (head injury[Title/Abstract])) OR (head trauma[Title/Abstract])) OR (decompressive craniectomy[Title/Abstract]) | 82053 |
|  | #16 OR #17 | 89405 |
|  | #7 AND #15 AND #18 | 304 |
| EmBase | | |
|  | 'randomized controlled trial'/exp | 848365 |
|  | 'controlled clinical trial'/exp | 1030429 |
|  | randomized:ab,ti | 1035608 |
|  | randomised:ab,ti | 205562 |
|  | randomly:ab,ti | 589771 |
|  | trial:ab,ti | 1215517 |
|  | groups:ab,ti | 3876482 |
|  | #1 OR #2 OR #3 OR #4 OR #5 OR #6 OR #7 | 5625626 |
|  | 'erythrocyte transfusion'/exp | 38408 |
|  | 'blood transfusion'/exp | 243432 |
|  | 'blood component therapy'/exp | 67773 |
|  | 'hemoglobin'/exp | 517366 |
|  | 'anemia'/exp | 530630 |
|  | anemia:ab,ti | 218254 |
|  | 'erythrocyte transfusion':ab,ti OR 'red blood cell transfusion':ab,ti OR 'blood transfusion':ab,ti OR 'blood component transfusion':ab,ti OR 'hemoglobin':ab,ti OR 'eryhem':ab,ti OR 'ferrous hemoglobin':ab,ti | 302701 |
|  | #9 OR #10 OR #11 OR #12 OR #13 OR #14 OR #15 | 1193902 |
|  | 'traumatic brain injury'/exp | 73535 |
|  | 'traumatic brain injuries':ab,ti OR 'brain trauma':ab,ti OR 'brain traumas':ab,ti OR 'traumatic brain injury':ab,ti OR 'traumatic encephalopathies':ab,ti OR 'traumatic encephalopathy':ab,ti OR 'head injury':ab,ti OR 'head trauma':ab,ti OR 'decompressive craniectomy':ab,ti | 109852 |
|  | #17 OR #18 | 125696 |
|  | #8 AND #16 AND #19 | 769 |
| COCHRANE CENTRAL | | |
|  | MeSH descriptor: [Erythrocyte Transfusion] explode all trees | 843 |
|  | MeSH descriptor: [Blood Transfusion] explode all trees | 4815 |
|  | MeSH descriptor: [Blood Component Transfusion] explode all trees | 1503 |
|  | MeSH descriptor: [Hemoglobins] explode all trees | 13574 |
|  | (Erythrocyte Transfusion):ti,ab,kw OR (Red Blood Cell Transfusion):ti,ab,kw OR (Blood Transfusion):ti,ab,kw OR (Blood Component Transfusion):ti,ab,kw OR (Hemoglobin):ti,ab,kw | 58583 |
|  | (Eryhem):ti,ab,kw OR (Ferrous Hemoglobin):ti,ab,kw | 985 |
|  | MeSH descriptor: [Anemia] explode all trees | 7442 |
|  | (Anemia):ti,ab,kw | 25997 |
|  | #1 OR #2 OR #3 OR #4 OR #5 OR #6 OR #7 OR #8 | 76917 |
|  | MeSH descriptor: [Brain Injuries, Traumatic] explode all trees | 1737 |
|  | (Traumatic Brain Injuries):ti,ab,kw OR (Brain Trauma):ti,ab,kw OR (Brain Traumas):ti,ab,kw OR (Traumatic Brain Injury):ti,ab,kw OR (Traumatic Encephalopathies):ti,ab,kw | 6598 |
|  | (Traumatic Encephalopathy):ti,ab,kw OR (head injury):ti,ab,kw OR (head trauma):ti,ab,kw OR (decompressive craniectomy):ti,ab,kw | 4809 |
|  | #10 OR #11 OR #12 | 10213 |
|  | #9 AND #13 | 350 |

Table S2. Sensitivity analysis of mortality.

|  | **NO. patients(trials)** | **RR** | **95%CI** | **I^2^** |
| --- | --- | --- | --- | --- |
| All trials | 1045 (4) | 1.00 | 0.80, 1.24 | 0% |
| Utilizing a fixed-effect model. | 1045 (4) | 1.00 | 0.80, 1.24 | 0% |
| Excluding trials with a weight less than 10% (McIntyre et al., 2006, Gobatto et al., 2019). | 934 (2) | 0.96 | 0.77, 1.21 | 0% |
| Excluding trials with Mclntyre et al., 2006 | 978 (3) | 1.02 | 0.70, 1.48 | 27% |
| Excluding trials with Turgeon et al., 2014 | 845 (3) | 1.16 | 0.70, 1.91 | 25% |
| Excluding trials with Gobatto et al., 2019 | 1001 (3) | 0.97 | 0.78, 1.21 | 0% |
| Excluding trials with Turgeon et al., 2024 | 311 (3) | 1.21 | 0.61, 2.41 | 29% |

RR: relative risk; Cl: confidence interval

Table S3. Sensitivity analysis of unfavorable neurological outcome.

|  | **NO. patients(trials)** | **RR** | **95%CI** | **I^2^** |
| --- | --- | --- | --- | --- |
| All trials | 1422 (4) | 1.06 | 0.94, 1.20 | 47% |
| Utilizing a fixed-effect model. | 1422 (4) | 1.08 | 1.00, 1.16 | 47% |
| Excluding trials with a weight less than 10% (Gobatto et al., 2019). | 1378 (3) | 1.07 | 0.99, 1.15 | 57% |
| Excluding trials with Robertson et al., 2014 | 1241 (3) | 1.11 | 1.03, 1.20 | 0% |
| Excluding trials with Gobatto et al., 2019 | 1378 (3) | 1.07 | 0.99, 1.15 | 57% |
| Excluding trials with Turgeon et al., 2024 | 700 (3) | 1.08 | 0.96, 1.21 | 64% |
| Excluding trials with Taccone et al., 2024 | 947 (3) | 1.04 | 0.96, 1.14 | 53% |

RR: relative risk; Cl: confidence interval

Figure S1. Risk of bias summary.


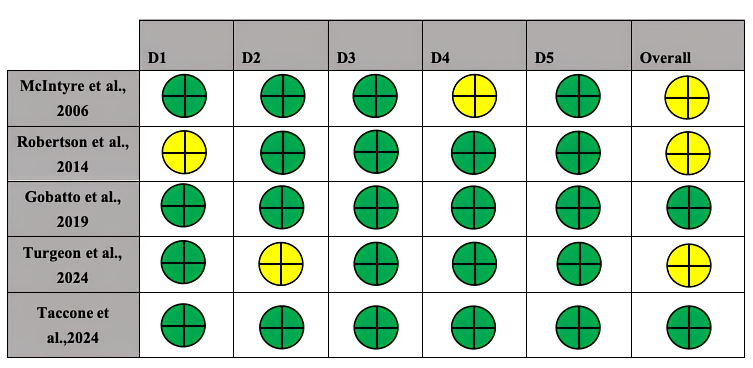
Domains:

D1: Bias arising from the randomization process.

D2: Bias due to deviations from intended intervention.

D3: Bias due to missing outcome data.

D4: Bias in measurement of the outcome.

D5: Bias in selection of the reported result.


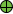
: Low risk of bias


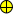
: some concerns risk of bias


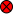
: High risk of bias

Figure S2. Subgroup analysis of the primary outcome.


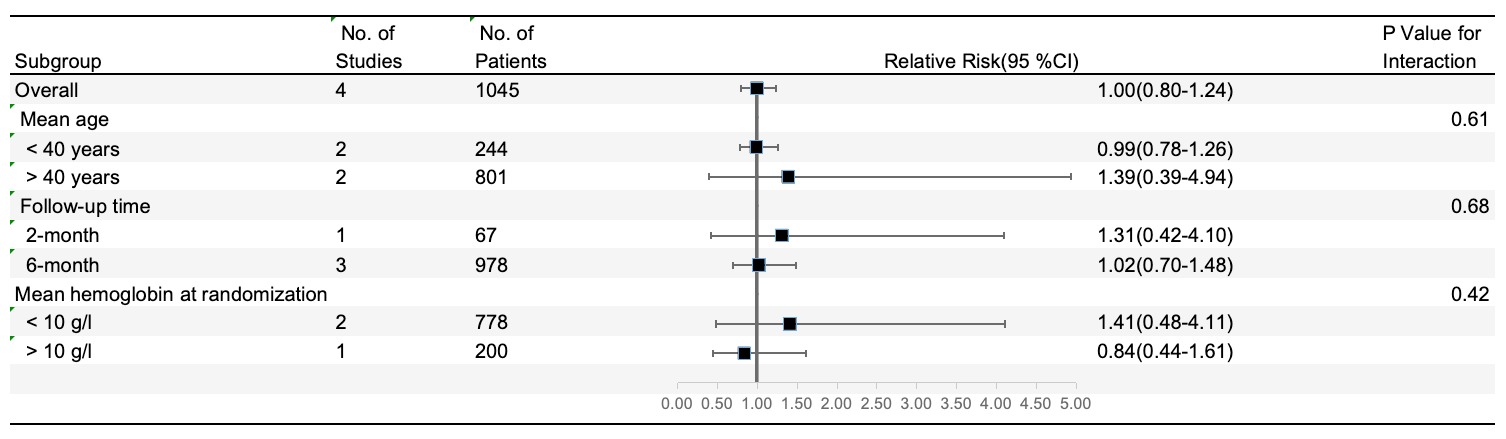

Supplement: Supplementary file 1 — Supplementary Material 1 [file 13613_2024_1411_MOESM1_ESM.docx]
